# Fully automated searching for the optimal VMAT jaw settings based on Eclipse Scripting Application Programming Interface (ESAPI) and RapidPlan knowledge‐based planning

**DOI:** 10.1002/acm2.12313

**Published:** 2018-03-25

**Authors:** Yuliang Huang, Haizhen Yue, Meijiao Wang, Sha Li, Jian Zhang, Zhuolun Liu, Yibao Zhang

**Affiliations:** ^1^ Key Laboratory of Carcinogenesis and Translational Research (Ministry of Education/Beijing) Department of Radiation Oncology Peking University Cancer Hospital & Institute Beijing Cancer Hospital & Institute Beijing Beijing China; ^2^ Department of Medical Physics School of Foundational Education & Institute of Medical Humanities Peking University Health Science Center Beijing China; ^3^ Beijing City Key Lab for Medical Physics and Engineering Institute of Heavy Ion Physics School of Physics Peking University Beijing China

**Keywords:** ESAPI, jaw optimization, RapidPlan, treatment planning, VMAT

## Abstract

**Purpose:**

Eclipse treatment planning system has not been able to optimize the jaw positions for Volumetric Modulated Arc Therapy (VMAT). The arbitrary and planner‐dependent jaw placements define the maximum field size within which multi‐leaf‐collimator (MLC) sequences can be optimized to modulate the beam. Considering the mechanical constraints of MLC transitional speed and range, suboptimal X jaw settings may impede the optimization or undermine the deliverability. This work searches optimal VMAT jaw settings automatically based on Eclipse Scripting Application Programming Interface (ESAPI) and RapidPlan knowledge‐based planning.

**Methods and materials:**

Using an ESAPI script, the X jaws of rectal VMAT plans were initially set to conform the planning‐target‐volume (PTV), and were gradually extended toward the isocenter (PTV center) in 5–7 mm increments. Using these jaw pairs, 592 plans were automatically created for 10 patients and quantitatively evaluated using a comprehensive scoring function. A published RapidPlan model was evoked by ESAPI to generate patient‐specific optimization objectives without manual intervention. All candidate plans were first stored as text files to save storage space, and only the best, worst, and conformal plans were consequently recreated for comparison.

**Results:**

Although RapidPlan estimates dose‐volume histogram (DVH) based on individual anatomy, the geometry‐based expected dose (GED) algorithm does not recognize different jaw settings but uses PTV‐conformal jaws as default; hence, identical DVHs were observed regardless of planner‐defined jaws. Therefore, ESAPI finalized dose‐volume calculation and eliminated the plans with unacceptable hotspots before comparison. The plan quality varied dramatically with different jaw settings. Trade‐offs among different organs‐at‐risk (OARs) were collectively considered by the proposed scoring method, which identified the best and worst plans correctly. The plans using conformal jaws were neither the best nor the worst of all candidates.

**Conclusions:**

VMAT plans using optimal jaw locations can be created automatically using ESAPI and RapidPlan. Conformal jaws are not the optimal choice.

## 1 INTRODUCTION

Although the in‐field beam intensity can be modulated by the optimizer,^1^ the jaw locations for VMAT are not optimized by the engine of Varian Eclipse treatment planning system.^2^ Even for jaw‐tracking technique, the planner‐defined values still determine the largest field size within which MLCs can modulate the beams. Tracking jaws are only programmed to reduce the low dose spillage outside MLC apertures, but are not optimized for finding the best MLC sequences.^3^ Limited by the physical constraints of MLCs such as translational range and speed, large jaw settings may impede the MLCs to reach the best location timely to shield the OAR,^4^ while small jaw size may induce inadequate target dose coverage. Optimal jaw settings may assist the optimizer to find better solutions^5^ which can be less challenging for MLC speeds and acceleration, hence increase the delivery accuracy.^6^ However, setting VMAT jaws has been a very arbitrary and planner‐dependent practice clinically, which might be more complex when the target dimension varies dramatically from different beams‐eye‐views.^7^

To explicitly display the dosimetric impact of jaw settings on the VMAT planning and find the best configuration, this work used ESAPI to create and evaluate a large number of plans automatically using various jaw settings, which can be hardly performed by a human before. RapidPlan knowledge‐based planning was also involved to minimize the planner dependence^8^, ^9^ and to automate the assignment of personalized optimization objectives for ten patients.^10^

## 2 MATERIALS AND METHODS

This study was performed on Varian Eclipse Treatment Planning System V. 13.6.

### 2.A ESAPI Scripting and plan creation

C#‐based plug‐in scripts were developed in an ESAPI research mode to duplicate and modify the parameters of historical VMAT plans for presurgical rectal cancer patients. The contouring, prescription, and planning protocols were based on Li's study^11^ and RTOG 0822 protocols.^12^ The plan was accessed through the “Context” interface in the “VMS.TPS” namespace. Information can be extracted from multiple data structures under the interface.

The plans were optimized with 10 MV photon, 1 full arc, and 5° collimator angle. The isocenter coincided with PTV center. Photon Optimizer v. 13.6 was used for the optimization. Initially, conformal X jaws to PTV border without margin were placed by the API, larger than which may increase unnecessary OAR exposure from MLC dose leakage. The Y jaws were further retracted by the width of an adjacent leaf of Millennium 120 MLCs for scatter contribution.^13^ Keeping all other parameters unchanged, plans with various X jaw sizes and positions were created by the scripts: One patient was used to test the method feasibility and display the dosimetric sensitivity to finer jaw changes, where the X jaws were gradually extended toward the isocenter by 50 mm (5 mm/step, 10 steps for each bank). The combined settings of two jaws yielded 100 possibilities. Nine more patients were optimized for statistical comparison but a larger step size (5–7 mm/step, 49–100 plans per patient) was used to accelerate the computation.

A predeveloped and validated RapidPlan model^14^, ^15^ was evoked by the script to automate and personalize the assignment of optimization objectives. However, identical optimization objectives were observed in all candidate plans for the same patient using different jaw openings, indicating RapidPlan does not use the actual jaw positions but conformal jaw coordinates for DVH estimation. This defect denies the predicted dose as a potentially faster indicator of plan quality change. Alternatively, an ESAPI script was used to duplicate the plan, change the jaw settings, optimize and calculate the volume dose for all candidate plans. However, storing all candidate plans could take too much space and time to open. Instead, only critical information was recorded as text files, including predicted DVH for OARs (to verify the independence of RapidPlan from actual jaw positions), final DVH calculations, and the corresponding jaw positions. Plans were deleted thereafter.

### 2.B Plan scoring and postprocessing

To ensure the target coverage, candidate plans were first normalized to meet the prescription before evaluating the OAR dose. Plans with >107% prescription hot spots were considered as clinically unacceptable and were removed before ranking. To simplify the collective consideration of all OAR dose indices, a plan scoring function was proposed to quantify the plan quality, whose values were calculated for each plan by postprocessing the text files using Python 3.5. The objective was to minimize the following score function where the subscript *i* refers to each OAR and *j* refers to each dose interval. The lower the plan score is, the better the OARs are spared.

$$score=\sum_{i}\frac{\left(MDVP_{i}+HDVP_{i}\right)}{\left(\bar{MDVP_{i}+HDVP_{i}}\right)}$$

where, $\text{MDVP}=\sum_{j}d_{j}\left(V_{d_{j}}-V_{d_{j+1}}\right)$and HDVP=∑jdj>didij(Vdj−Vdj+1)

MDVP stands for mean dose volume product, aiming to reduce the OAR mean dose. HDVP stands for integrated high dose volume product, aiming to minimize the high dose region^16^ as a combination of the multiple dose‐volume constraints per RTOG 0822 protocols. For small bowel, femoral head, and bladder, the HDVP were calculated for volumes receiving dose above 35, 40, and 40 Gy, respectively. Note that the subscript *i* in the first equation represents each OAR, while the subscript *j* in the following equations refers to discrete sampling dose points in the DVH curves. *d*_*j*_ *>d*_*t*_ means that summation is only done in high dose region (greater than a threshold dose level suggested by RTOG 0822). To evaluate the magnitude of absolute dose change on an organ‐specific basis, the normalization to the mean value of 100 plans (${\bar{MDVP_{i}+HDVP}}_{i}$) was conducted for each OAR to generate relative evaluators. For simplicity, equal weight was assigned to HDVP and MDVP.

Using the jaw positions recorded in the text files, the best, worst, and conformal plans were reproduced by the script for comparison to validate the jaw optimization and scoring function. Plotting was conducted using Matplotlib library.

To compare the dosimetrics of the best and conformal plans, paired *t*‐tests were conducted in terms of average dose and other significant dosimetric index according to RTOG 0822 of three OARs.

## 3 RESULTS

### 3.A Independence of RapidPlan from actual jaw settings

The exported RapidPlan‐estimated DVHs displayed no difference under various jaw settings, proving the independence of RapidPlan prediction from actual jaw settings. Therefore, final volume dose calculation was necessary for all candidate plans for comparison, which roughly cost half an hour per plan using AAA algorithm based on a standalone Eclipse workstation (Intel (R) Xeon (R) CUP E5‐2650 v4 @ 2.20 GHz 2.20GHz, 32.0 GB RAM).

### 3.B Plan quality under various jaw settings

**Figure 1:**
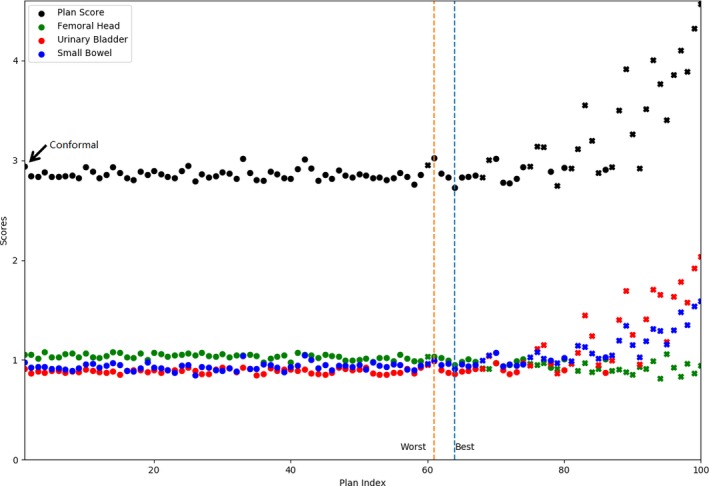
Plan scores and individual OAR scores of 100 candidate plans for the first patient using various jaw settings. The 26 clinically unacceptable plans with hot spots receiving over 107% of dose prescription are marked as “x”. The vertical dashed lines indicate the worst plan (orange, highest plan score = 3.02) and the best plan (blue, lowest plan score = 2.73), respectively. The conformal plan is the first plan on the left pointed by an arrow (plan score = 2.94).

For the first patient, the candidate plans were labeled consecutively from index 1 to 100. The plan scores as well as the individual OAR scores were plotted in Fig. 1. Twenty‐six plans were identified as clinically unacceptable due to hotspot and were marked as “x”. The vertical dashed blue and orange lines mark the best (lowest score = 2.73) and the worst plans (highest score = 3.02), respectively, of the remaining 74 plans. The plan using conformal jaws was plotted as the first one on the left (plan score = 2.94).

**Figure 2:**
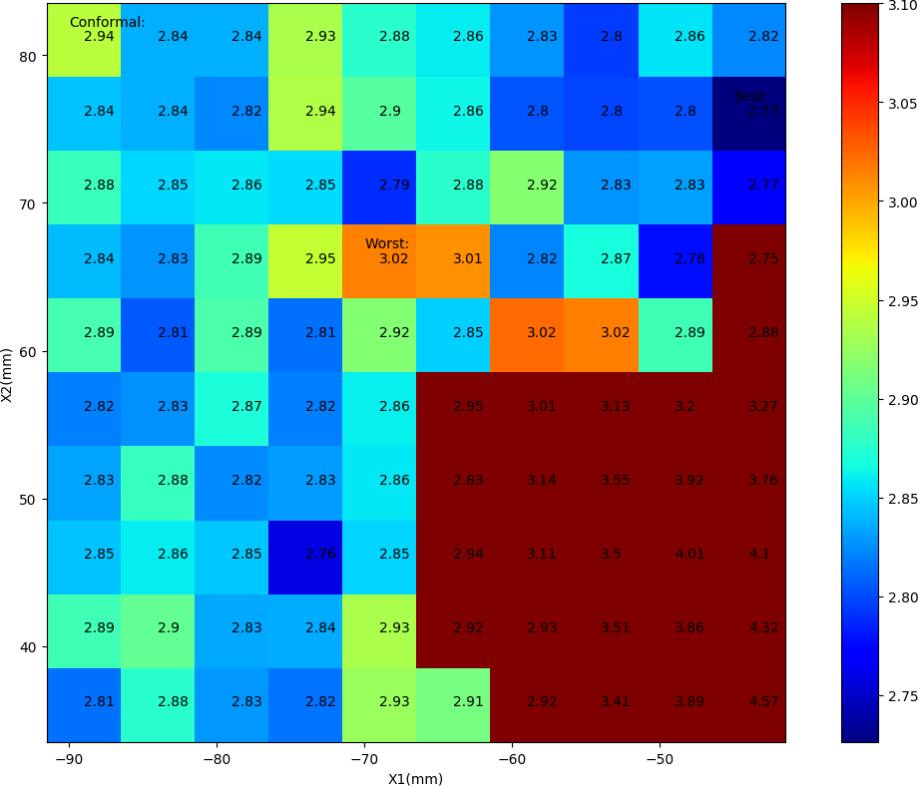
A heat map reflecting the sensitivity of plan scores to the X jaw sizes and locations for the first patient. Colder pixels indicate better OAR sparing, and the 26 clinically unacceptable cases were colored as uniform dark red. Increased absolute values of X1 and X2 axes indicate jaw retraction hence larger field sizes.

Using the first patient as an example, Fig. 2 displays the dependence of plan quality on the X jaw sizes and locations as a heat map, with plans’ score values labeled on the corresponding pixels. The best, worst, and conformal plans were also marked on the map. To better differentiate the pixels with close colors, the 26 clinically unacceptable plans were painted as uniform dark red. The colder color indicates lower plan score and better plan quality. The increased absolute values of X1 and X2 axes indicate jaw retraction, hence enlarged jaw opening.

**Figure 3:**
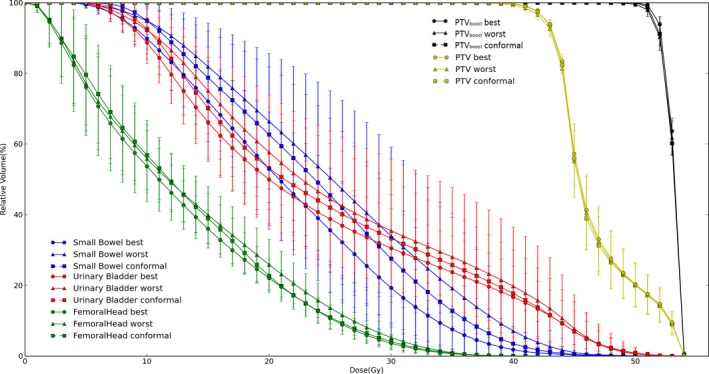
Mean DVHs of 10 patients comparing the best (circle), worst (triangle), and conformal (square) plans as suggested by the scores. The urinary bladder, small bowel, and femoral head are indicated by red, blue, and green colors, respectively. The error bars indicate 1 standard deviation.

The average DVHs of ten patients displaying the best, worst, and conformal plans as identified by the scoring function were plotted for comparison in Fig. 3, where the error bars indicate 1 standard deviation (SD). Note that multiple plans of equally high score may exist, wherein one of them was randomly selected for calculating the average DVHs in Fig. 3 to demonstrate the agreement between the plan score and ultimate DVHs.

Table 1 shows the statistics of the dosimetric metrics of all 100 candidate plans for the first patient. Table 2 compares the best, worst, and conformal jaw settings among 10 patients.

**Table 1:** Dosimetric statistics of 100 candidate plans using various jaw settings

	Minimum	Maximum	Mean ± SD	Best^a^	Worst^a^	Conformal
Small bowel						
MDVP	1721.50	2587.03	1963.71 ± 175.12	1845.54	2001.87	1924.78
HDVP	14.06	666.57	84.51 ± 118.07	28.57	37.86	75.18
Score	0.85	1.59	1.00 ± 0.14	0.91	0.99	0.98
Femoral head						
MDVP	898.52	1187.79	1098.62 ± 66.26	1044.37	1140.44	1160.59
HDVP	0	17.24	1.09 ± 2.71	2.50	0	0
Score	0.82	1.08	1.00 ± 0.06	0.95	1.04	1.06
Urinary bladder						
MDVP	1827.79	3523.41	2100.14 ± 371.26	1850.16	2161.90	1981.67
HDVP	345.38	1761.69	496.66 ± 286.81	380.63	409.56	380.63
Score	0.85	2.04	1.00 ± 0.25	0.86	0.99	0.91

a Excluding 26 unacceptable plans.

Abbreviations: MDVP, mean dose volume product; HDVP, high dose volume product, where for small bowel, femoral head, and urinary bladder, the HDVP were calculated for the volumes receiving no less than 35, 40, and 40 Gy, respectively, per RTOG 0822 protocols.

**Table 2:** Mean values ± 1SD of 10 patients’ best, worst, and conformal plans

	Best	Worst	Conformal	*P*
Small bowel				
V_35_	2.8 ± 2.5	7.4 ± 7.9	4.9 ± 4.2	0.009
V_40_	0.8 ± 0.9	3.0 ± 4.0	1.5 ± 2.1	0.126
V_45_	0.1 ± 0.3	0.4 ± 1.1	0.2 ± 0.5	0.638
$\bar{D}$	20.9 ± 3.5	24.7 ± 5.7	23.3 ± 4.5	0.003
Femoral head				
V_40_	0.2 ± 0.2	0.3 ± 0.3	0.3 ± 0.2	0.130
$\bar{D}$	12.1 ± 1.5	13.0 ± 1.9	12.7 ± 1.6	0.018
Urinary bladder				
V_40_	7.4 ± 4.0	8.7 ± 7.5	7.8 ± 5.6	0.523
V_45_	3.2 ± 2.7	3.6 ± 4.5	3.3 ± 4.0	0.980
V_50_	0.4 ± 0.7	0.3 ± 0.4	0.4 ± 0.8	0.905
$\bar{D}$	23.1 ± 3.8	25.0 ± 5.8	24.1 ± 5.2	0.102

Abbreviations: V_D_ means relative volume (%) receiving dose no less than D Gy. $\bar{D}$ means mean dose (Gy). P represents the *P*‐value of paired comparison between the best and conformal plans. Other RTOG 0822 recommended metrics <0.1 were not reported.

## 4 DISCUSSION

Although the RapidPlan model generated identical optimization objectives for the same patient anatomy and beam geometry (except jaws), the knowledge‐based planning module in the proposed optimal jaw searching method is intended to avoid subjective planner dependence, and to personalize the automated optimization in case of different patient anatomy, prescription, field geometry and energies, which were all modeled by RapidPlan in dose prediction.^9^, ^17^, ^18^, ^19^, ^20^ However, it is highly desired that, the next versions of RapidPlan should model the actual jaw settings for more accurate dose estimation, which may potentially serve as a fast and sensitive indicator of dosimetric changes with various jaw settings. Less than one twentieth time consumption can be anticipated that way since DVH estimation roughly takes less than 1 min (vs. ~30 min to finalize a plan).

The macroscopic dosimetric fluctuations as shown in Figs. 1 and 2; Table 1 confirm the sensitivity of VMAT plan quality to the jaw settings. The inter‐competition of OARs in the same plan can be interpreted from Fig. 1: the decreased dose to one OAR is often at cost of increased dose to another. It is unlikely to find a solution to achieve the minimum dose simultaneously for all OARs. That is why the individual OAR dose metrics of the best plan were consistently higher than the minimum values of 100 plans (Table 1). The best plan struck a balance through evaluating various OAR dose indices collectively, by means of a scoring function in this study. As a reminder, the components and weighting factors of the scoring function can be adjusted to comply with the site‐specific OARs, other institutionally preferred trade‐offs or clinical protocols. Similar score functions may also be used for automatic QA purposes using ESAPI.

Plans with hotspot >107% of prescription were excluded per our clinical preference and ICRU 83 protocols.^21^ The over‐shrunk jaw‐induced target under‐dose, and the hot spots were amplified after normalization to prescription.

On the basis of largely overlapping DVHs of the targets, Fig. 3 suggests that the plan quality can be well reflected by the proposed plan scoring function. Figure 3 also demonstrates that conformal jaws are not necessarily the optimal setting for VMAT planning, agreeing with the dosimetric comparison in Table 2. Results of paired T‐test showed that all optimal plans displayed lower (4 out of 10 DVH metrics displayed statistical significance) or at least equal OAR dose metrics than the conformal jaw settings.

Potentially, the proposed method can be improved in a few aspects: (a) A standalone ESAPI script can be created for batch processing multiple plans without opening individual cases; (b) The searching interval of 5 mm can be increased further for higher efficiency, or decreased for possibly better solutions. (c) Considering that multiple cold pixels were observed in Fig. 2, several good candidates can be proposed for clinical selection to further evaluate the monitor units (modulation complexity) or pretreatment QA performance for instance. These concerns could also be incorporated to the scoring function for automation. In addition, optimal settings for other parameters can be searched automatically using the proposed method, such as collimator angle, beam angle, isocenter, beam energy, etc., which will be studied in the future. (d) Because a knowledge‐based model learn features from its training cohort, the generated plan optimization objectives may be more favorable to the jaw settings that were similar to the training plans. Further studies are desirable to investigate these unpredicted uncertainties.

## 5 CONCLUSIONS

VMAT plans using optimal jaw settings can be created automatically using Eclipse Scripting Application Programming Interface and RapidPlan knowledge‐based planning. Suboptimal or even unqualified plans are associated with conformal or arbitrary jaw definitions.

## CONFLICT OF INTEREST

Dr. Yibao Zhang received speaker's honorarium from Varian Medical Systems. This work was partially supported by Varian Research Collaboration Grant (Strategies for RapidPlan Model Adaptation).

## References

[1] Vanetti E , Nicolini G , Nord J , et al. On the role of the optimization algorithm of RapidArc volumetric modulated arc therapy on plan quality and efficiency. Med Phys. 2011;38:5844–5856. 22047348 10.1118/1.3641866

[2] Cozzi L , Dinshaw KA , Shrivastava SK , et al. A treatment planning study comparing volumetric arc modulation with RapidArc and fixed field IMRT for cervix uteri radiotherapy. Radiother Oncol. 2008;89:180–191. 18692929 10.1016/j.radonc.2008.06.013

[3] Wu H , Jiang F , Yue H , et al. A comparative study of identical VMAT plans with and without jaw tracking technique. J Appl Clin Med Phys. 2016;17:133–141. 27685122 10.1120/jacmp.v17i5.6252 PMC5874095

[4] Huang B , Fang Z , Huang Y , et al. A dosimetric analysis of volumetric‐modulated arc radiotherapy with jaw width restriction vs 7 field intensity‐modulated radiotherapy for definitive treatment of cervical cancer. Br J Radiol. 1039;2014:20140183. 10.1259/bjr.20140183 PMC4075592 24834477

[5] Mancosu P , Navarria P , Castagna L , et al. Anatomy driven optimization strategy for total marrow irradiation with a volumetric modulated arc therapy technique. J Appl Clin Med Phys. 2012;13:138–147. 10.1120/jacmp.v13i1.3653 PMC5716136 22231216

[6] Park JM , Wu HG , Kim JH , et al. The effect of MLC speed and acceleration on the plan delivery accuracy of VMAT. Br J Radiol. 1049;2015:20140698. 10.1259/bjr.20140698 PMC4628477 25734490

[7] Wu Q , Chin SK , Chang L , et al. Optimization of treatment geometry to reduce normal brain dose in radiosurgery of multiple brain metastases with single‐isocenter volumetric modulated arc therapy. Sci Rep. 2016;6:34511. 27688047 10.1038/srep34511 PMC5043272

[8] Lian J , Yuan L , Ge Y , et al. Modeling the dosimetry of organ‐at‐risk in head and neck IMRT planning: an inter technique and inter institutional study. Med Phys. 2013;40:121704. 24320490 10.1118/1.4828788 PMC3838428

[9] Berry SL , Ma R , Boczkowski A , et al. Evaluating inter‐campus plan consistency using a knowledge based planning model. Radiother Oncol. 2016;120:349–355. 27394695 10.1016/j.radonc.2016.06.010 PMC5003669

[10] Wu B , Ricchetti F , Sanguineti G , et al. Data‐driven approach to generating achievable dose‐volume histogram objectives in intensity‐modulated radiotherapy planning. Int J Radiat Oncol Biol Phys. 2011;79:1241–1247. 20800382 10.1016/j.ijrobp.2010.05.026

[11] Li J , Ji J , Cai Y , et al. Preoperative concomitant boost intensity‐modulated radiotherapy with oral capecitabine in locally advanced mid‐low rectal cancer: a phase II trial. Radiother Oncol. 2012;102:4–9. 21903285 10.1016/j.radonc.2011.07.030

[12] Garofalo M , Moughan J , Hong T , et al. RTOG 0822: a Phase II Study of Preoperative (PREOP) Chemoradiotherapy (CRT) Utilizing IMRT in Combination with Capecitabine (C) and Oxaliplatin (O) for Patients with Locally Advanced Rectal Cancer. Int J Radiat Oncol Biol Phys. 2011;81:S3–S4. 10.1016/j.ijrobp.2015.05.005 PMC4540628 26163334

[13] Appenzoller LM , Michalski JM , Thorstad WL , et al. Predicting dose‐volume histograms for organs‐at‐risk in IMRT planning. Med Phys. 2012;39:7446–7461. 23231294 10.1118/1.4761864

[14] Hao W , Fan J , Yue H , et al. Applying a RapidPlan model trained on a technique and orientation to another: a feasibility and dosimetric evaluation. Radiat Oncol. 2016;11:108. 27538431 10.1186/s13014-016-0684-9 PMC4990878

[15] Wu H , Jiang F , Yue H , et al. A dosimetric evaluation of knowledge‐based VMAT planning with simultaneous integrated boosting for rectal cancer patients. J Appl Clin Med Phys. 2016;17:78–85. 27929483 10.1120/jacmp.v17i6.6410 PMC5690500

[16] Salter BJ , Fuss M , Sarkar V , et al. Optimization of isocenter location for intensity modulated stereotactic treatment of small intracranial targets. Int J Radiat Oncol Biol Phys. 2009;73:546–555. 19147019 10.1016/j.ijrobp.2008.09.011

[17] Tol JP , Delaney AR , Dahele M , et al. Evaluation of a knowledge‐based planning solution for head and neck cancer. Int J Radiat Oncol Biol Phys. 2015;91:612–620. 25680603 10.1016/j.ijrobp.2014.11.014

[18] Fogliata A , Belosi F , Clivio A , et al. On the pre‐clinical validation of a commercial model‐based optimisation engine: application to volumetric modulated arc therapy for patients with lung or prostate cancer. Radiother Oncol. 2014;113:385–391. 25465726 10.1016/j.radonc.2014.11.009

[19] Fogliata A , Nicolini G , Bourgier C , et al. Performance of a knowledge‐based model for optimization of volumetric modulated arc therapy plans for single and bilateral breast irradiation. PLoS ONE. 2015;10:e0145137. 26691687 10.1371/journal.pone.0145137 PMC4686991

[20] Fogliata A , Nicolini G , Clivio A , et al. A broad scope knowledge based model for optimization of VMAT in esophageal cancer: validation and assessment of plan quality among different treatment centers. Radiat Oncol. 2015;10:220. 26521015 10.1186/s13014-015-0530-5 PMC4628288

[21] ICRU Report 83 . Prescribing, recording, and reporting photon‐beam intensity‐modulated radiation therapy (IMRT). J ICRU. 2010;10:1–106. 10.1007/s00066-011-0015-x 22234506

